# hnRNP A1 inhibits colorectal cancer tumorigenesis and progression by regulating fatty acid metabolism and RNA stability

**DOI:** 10.1038/s41420-025-02814-0

**Published:** 2025-11-24

**Authors:** Kai Ji, Leqi Zhou, Tianshuai Zhang, Hao Fan, Gang Xie, Wenjiang Man, Can Wang, Yucheng Tian, Li Chen, Guimin Wang, Mulin Liu, Bing Zhu, Wei Zhang, Guanyu Yu

**Affiliations:** 1 Department of Colorectal Surgery, Shanghai Changhai Hospital, Naval Medical University, Shanghai, China; 2 Department of Gastrointestinal Surgery, The First Affiliated Hospital of Bengbu Medical University, Bengbu, Anhui China; 3 Department of Anorectal Surgery, Huaibei People’s Hospital, Huaibei, Anhui China

**Keywords:** Colorectal cancer, Cell growth, Cell migration

## Abstract

Increasing evidence indicates that RNA-binding proteins and the reprogramming of lipid metabolism play crucial roles in tumorigenesis. However, the extent to which members of these families contribute, and whether targeting metabolic genes to affect overall protein production in cancer cells, remains largely unknown. This study analyzes CRC tissue samples and databases to reveal the high expression of hnRNP A1 in CRC and its critical role in tumorigenesis, proliferation, migration, and prognosis. Through combined sequencing analyses, we identified a novel mechanism by which PPARα regulates lipid metabolism. Our data indicate that hnRNP A1 is central to lipid metabolism reprogramming in CRC, promoting lipid accumulation by regulating PPARα mRNA stability, thereby influencing cell proliferation and apoptosis. Overall, hnRNP A1 could serve as a novel target for CRC therapy. Its involvement in cancer development offers new biological insights and potential therapeutic strategies. As a potential biomarker and therapeutic target, it presents novel approaches for the clinical management of CRC.

## Introduction

Colorectal cancer (CRC) is the third most common cancer and the second leading cause of cancer-related deaths worldwide, posing a major threat to human health [1, 2]. CRC is characterized by its marked invasiveness and metastatic potential. Tumor recurrence and metastasis are key contributors to poor prognosis in CRC patients, significantly reducing their quality of life [3, 4]. Therefore, it is essential to investigate the molecular mechanisms driving metastasis and conduct extensive research to identify and validate specific biomarkers and therapeutic targets for CRC management.

The hnRNP family plays a pivotal role in tumorigenesis and progression, influencing processes such as inflammation, immunity, angiogenesis, gene expression, apoptosis, and cancer cell invasion and metastasis [5–10]. hnRNP proteins are also implicated in DNA maintenance, subcellular localization, and the regulation of mRNA translation and stability [11–13]. hnRNP A1 is a key component that regulates the synthesis and translation of tumor-associated proteins, contributing to RNA metabolism, nucleocytoplasmic transport, alternative splicing, and telomere elongation, thus promoting tumor cell proliferation [14–16]. A substantial body of experimental evidence indicates that hnRNP A1 is overexpressed in various cancers, including lung, thyroid, bladder, and breast cancers, where it regulates tumorigenesis through multiple mechanisms [10, 17–19]. Upregulation of hnRNP A1 serves as an independent predictor of early biochemical recurrence in prostate cancer [20]. Other studies suggest that hnRNP A1 may serve as a potential biomarker for the early diagnosis, prognostic assessment, and disease monitoring of CRC [21, 22]. However, the specific role of hnRNP A1 in CRC and its underlying molecular mechanisms remain inadequately studied, necessitating further exploration to better understand its function in CRC initiation and progression.

Abnormal alterations in energy metabolism during cancer initiation, progression, and treatment resistance have gained widespread recognition in recent years [23, 24]. The relationship between energy metabolism and cancer initiation is complex, involving multiple cellular metabolic pathways and physiological mechanisms [25]. Cancer cells reprogram their metabolism to meet the energy and material demands of rapid proliferation. This metabolic reprogramming, referred to as “tumor metabolism reprogramming,” is a hallmark of cancer initiation and progression [26, 27]. Disruption of lipid metabolism is recognized as a key metabolic hallmark of cancer [28–31]. Moreover, lipid metabolism reprogramming is closely associated with cellular interactions in the tumor microenvironment. Lipid metabolism reprogramming allows cancer cells to acquire both structural and energy sources, while also activating multiple pro-cancer signaling pathways via lipid signaling molecules [32]. Lipid metabolism also plays a crucial role in cancer cell adaptation to the microenvironment, immune evasion, and the antioxidant stress response. By releasing and absorbing lipid molecules, such as fatty acids, cancer cells modulate the function of surrounding stromal, immune, and endothelial cells, further promoting tumor proliferation, angiogenesis, and metastasis. As a result, lipid metabolism has become a key target for cancer therapy [33–36]. CRC development involves complex metabolic changes and interactions with the microenvironment. However, research on lipid metabolism in CRC remains limited, underscoring the need for further in-depth exploration.

We initially observed that hnRNP A1 is upregulated in CRC and plays a critical role in the growth and metastasis of cancer cells. Using sequencing and other techniques, we identified peroxisome proliferator-activated receptor alpha (PPARα) as a direct target of hnRNP A1 in CRC cells. In vitro studies were conducted to investigate the biological function of PPARα in CRC and its potential regulation of fatty acid (FA) metabolism, further contributing to its role in CRC. We found that hnRNP A1 regulates PPARα translation by controlling its mRNA stability, which in turn affects lipid metabolism in CRC cells. Our data thus demonstrate the critical role of the hnRNP A1/PPARα axis in cancer progression.

## Results

### hnRNPA1 is upregulated in CRC and is associated with poor prognosis

**Fig. 1:**
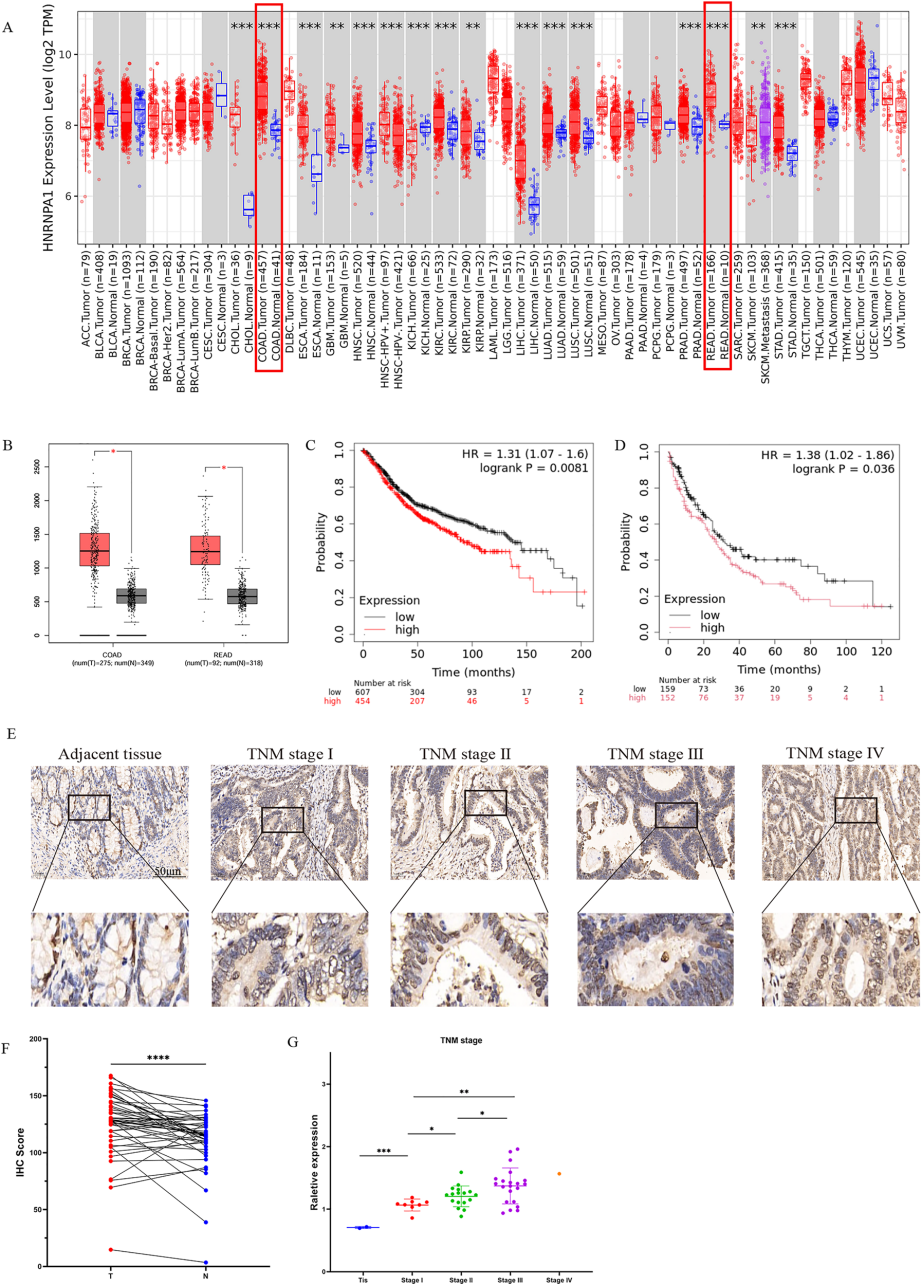
hnRNP A1 is upregulated in CRC tissue, and high expression of hnRNP A1 is associated with poor prognosis. **A** Expression of hnRNP A1 in different tumors. **B** Expression of hnRNP A1 in colorectal cancer. **C** Overall survival of colorectal cancer patients. **D** Post progression Survival in colorectal cancer patients. **E** Immunohistochemical staining of colorectal cancer and adjacent tissues (Scale bar: 50 μm). **F** Immunohistochemical staining scoring. **G** Expression of hnRNP A1 in CRC basedon individual cancer stages. *P < 0.05, **P < 0.01, ***P < 0.001, ****P < 0.0001.

RNA-binding proteins (RBPs) are essential in multiple stages of RNA processing, including splicing, maturation, transport, translation regulation, and degradation. hnRNP family proteins are key members of RBPs and play a central role in the complex regulation of gene expression, essential for the function and development of normal cells. A comprehensive analysis of hnRNP family members’ expression levels in CRC was performed using the TCGA database. The results revealed that hnRNP A1 expression was significantly higher than that of other hnRNP family members, and was notably elevated in tumor tissue compared to normal tissue (Supplementary Fig. S1A). A pan-cancer analysis of hnRNP A1 expression was conducted using the TCGA dataset to examine its expression across different cancer types. The results indicated that hnRNP A1 expression was notably elevated in cancers of the digestive system, including CRC, cholangiocarcinoma, esophageal cancer, and gastric cancer (Fig. 1A). Finally, a detailed analysis of CRC confirmed that hnRNP A1 expression was significantly higher in tumor tissue than in normal tissue (Fig. 1B). Based on these findings, hnRNP A1 may function as an oncogene, promoting tumorigenesis and progression. To evaluate the impact of hnRNP A1 expression on the prognosis of CRC, survival analysis was conducted using a public colorectal cancer dataset (kmplot.com). The results indicated that patients with high hnRNP A1 expression had significantly shorter overall survival (OS) and post-progression survival (PPS) compared to those with low expression (Fig. 1C, D). Furthermore, high expression of hnRNP family members was negatively correlated with overall survival (OS) in CRC patients (Supplementary Fig. S1B–K). These findings suggest that hnRNP family proteins may play an oncogenic role in CRC, with high expression serving as a marker of poor prognosis. To validate these findings, tissue microarray analysis and immunohistochemical scoring were performed on samples from 116 postoperative CRC patients in the colorectal surgery department of Changhai Hospital. The results were consistent with those from the TCGA dataset, showing that hnRNP A1 expression was significantly higher in CRC tissue than in normal tissue (Fig. 1E, F). Additionally, patients were evaluated based on TNM staging (I-IV), and protein expression was analyzed at each stage. hnRNP A1 expression was closely associated with TNM stage, being significantly higher in stages II-III compared to stage I (Fig. 1G). These results suggest that hnRNP A1 is significantly overexpressed in CRC and is strongly associated with poor prognosis.

### hnRNP A1 enhances the proliferation and migration of CRC cells

**Fig. 2:**
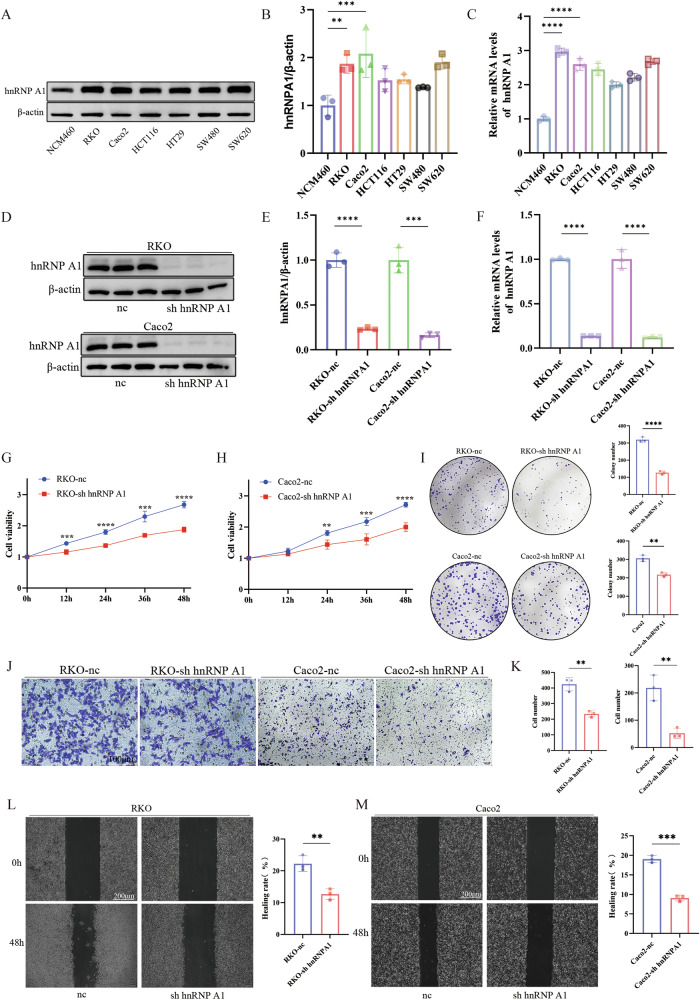
hnRNP A1 enhances the proliferation and migration of CRC cells. **A**, **B** hnRNP A1 expression levels in six CRC cell lines and NCM460 determined by Western blotting, *n* = 3 per group. **C** hnRNP A1 expression levels in six CRC cell lines and NCM460 determined by qRT-PCR, *n* = 3 per group. **D**, **E** Western blotting verify the shRNA-mediated deletion of hnRNP A1 in RKO and Caco2 cells, *n* = 3 per group. **F** qRT-PCR verify the shRNA-mediated deletion of hnRNP A1 in RKO and Caco2 cells, *n* = 3 per group. **G**, **H** CCK-8 assay for assessing cell proliferation, *n* = 6 per group. **I** The proliferation ability of the cells was verified by clonal formation assay, *n* = 3 per group. **J**, **K** Transwell assay for assessing cell migration(Scale bar: 100 μm), *n* = 3 per group. **L**, **M** Wound-healing assay for assessing cell migration(Scale bar: 200 μm), *n* = 5 per group. **P < 0.01, ***P < 0.001, ****P < 0.0001.

Baseline mRNA and protein expression levels of hnRNP A1 were evaluated in six CRC cell lines and the normal colonic epithelial cell line NCM460. hnRNP A1 was generally overexpressed in CRC cell lines compared to the normal control, with relatively high levels observed in RKO, Caco2, and SW620 cells, and lower levels in SW480, HT29, and HCT116 cells. (Fig. 2A–C). To evaluate the role of hnRNP A1 in CRC cell growth and metastasis, hnRNP A1 was knocked down in RKO and Caco2 cells, and the knockdown efficiency was validated. (Fig. 2D–F). CCK-8 assays and colony formation experiments revealed that hnRNP A1 knockdown significantly reduced cell proliferation and colony formation (Fig. 2G–I). Transwell and wound healing assays demonstrated that hnRNP A1 knockdown reduced cell migration (Fig. 2J–M). VPC-80051 is a small molecule inhibitor of hnRNP A1 that binds to the UP1 domain, reducing cell viability [37]. To evaluate its potential in CRC treatment, CRC cells (RKO, Caco2) were treated with different concentrations of VPC-80051. The results demonstrated a dose- and time-dependent decrease in cell viability, highlighting the therapeutic potential of VPC-80051 in CRC treatment (Supplementary Fig. S2A–C). These results suggest that hnRNP A1 is essential for CRC cell growth and migration, and its high expression may promote tumor proliferation and metastasis.

### Knocking down hnRNP A1 expression inhibits CRC tumorigenesis

**Fig. 3:**
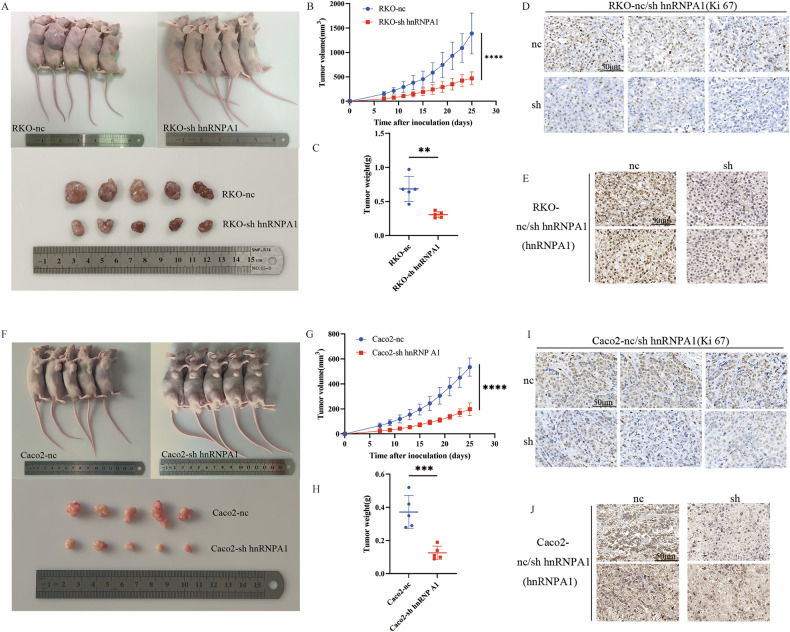
Knocking down hnRNP A1 expression inhibits CRC tumorigenesis. **A**–**C**, **F**–**H** Subcutaneous xenograft tumor images and tumor growth of RKO and Caco2 cells with hnRNP A1-nc and hnRNP A1-sh in nude mice, *n* = 5 per group. **D**, **E**, **I**, **J** The expressions of Ki-67 and hnRNP A1 in hnRNP A1-nc and hnRNP A1-sh xenografts were analyzed by IHC(Scale bar: 50 μm). **P < 0.01, ***P < 0.001, ****P < 0.0001.

To investigate whether hnRNP A1 promotes tumorigenesis, a tumor-bearing model was constructed to assess its potential in tumor development. RKO and Caco2 cells with hnRNP A1 knockdown (sh hnRNP A1) or control (nc) were subcutaneously injected into the axillae of nude mice. After 4 weeks, the mice were euthanized, and the tumors were collected (Fig. 3A, F). The results showed that the hnRNP A1 knockdown group exhibited slower subcutaneous tumor growth and smaller tumor volumes (Fig. 3B, G). Additionally, compared to the control group, the hnRNP A1 knockdown mice had significantly lower tumor weights (Fig. 3C, H). Furthermore, we evaluated the cell proliferation marker Ki-67 and hnRNP A1 expression in these tumors. Compared to control tumors, the hnRNP A1 knockdown tumors showed reduced hnRNP A1 expression and a significant decrease in Ki-67 positivity (Fig. 3D, E, I, J). In conclusion, these findings confirm that hnRNP A1 acts as an oncogene in CRC, promoting cell proliferation.

### hnRNP A1 can inhibit cell apoptosis and bind to PPARα

**Fig. 4:**
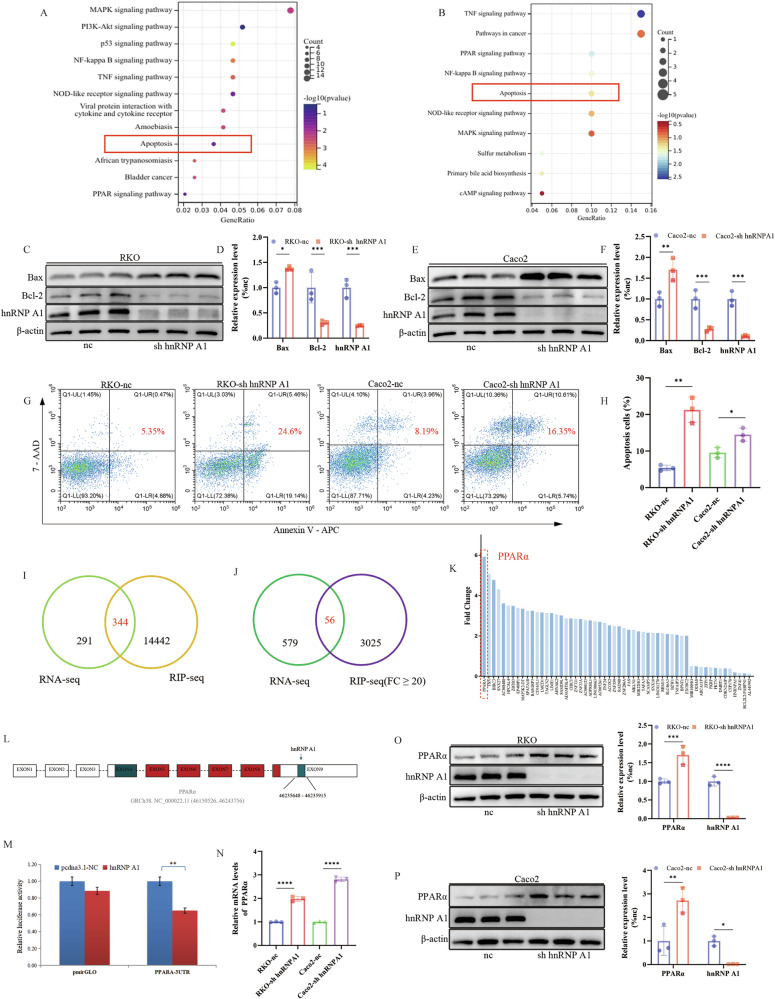
hnRNP A1 can inhibit cell apoptosis and bind to PPARα. **A** The mRNA with differential expression in RKO-nc and RKO-sh hnRNP A1 cells was analyzed by the KEGG pathway after RNA sequencing. **B** The KEGG pathway was analyzed after the hnRNP A1-binding protein was analyzed by RIP sequencing. **C**–**F** Western blotting demonstrates the expression changes of Bax and Bcl-2 in hnRNP A1-silenced cells, *n* = 3 per group. **G**, **H** The total apoptotic cells with the treatment of Adarotene for 24 h in RKO and Caco2 cells, *n* = 3 per group. **I**, **J** The number of genes shared after the intersection of RNA-seq and RIP-seq. **K** Fold change of 56 genes after screening. **L** Predicted hnRNP A1 and PPARα binding sites identified by RIP-seq. **M** A dual-luciferase reporter assay was used to assess the interaction between hnRNP A1 and the PPARα 3′UTR, *n* = 3 per group. **N** PPARα expression levels in CRC cell lines determined by qRT-PCR, *n* = 3 per group. **O**, **P** PPARα expression levels in CRC cell lines determined by Western blotting, *n* = 3 per group.*P < 0.05, **P < 0.01, ***P < 0.001, ****P < 0.0001.

**Fig. 5:**
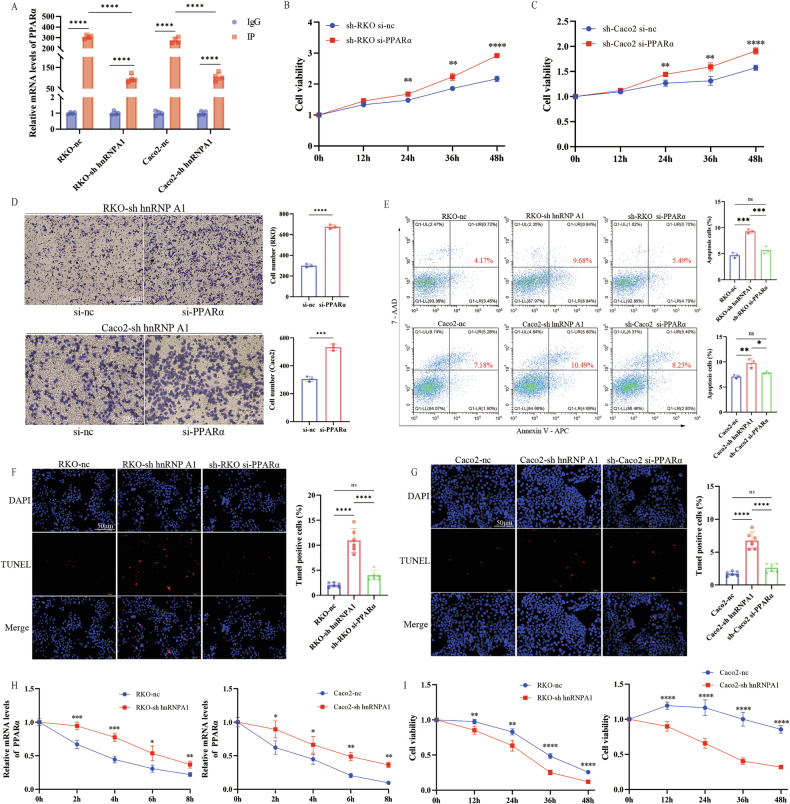
Knockdown of PPARα rescued the proliferation and migration of colorectal cancer cells. **A** The binding level of hnRNPA1 to PPARα in CRC cell lines was determined by RIP, *n* = 4 per group. **B**, **C** CCK-8 assay was used to determine cell proliferation after interference with PPARα gene, *n* = 6 per group. **D** The Transwell assay assesses the migration ability of cells after interference with the PPARα gene (Scale bar: 100 μm), *n* = 3 per group. **E** The number of apoptotic cells in RKO and Caco2 cells after PPARα gene interference was determined by flow cytometry, *n* = 3 per group. **F**, **G** The number of apoptotic cells in RKO and Caco2 cells after PPARα gene interference was determined by TUNEL (Scale bar: 50 μm), *n* = 6 per group. **H** The expression level of PPARα in cells treated with Act D at different time periods, *n* = 3 per group. **I** Cell activity after treatment with Act D, *n* = 6 per group. *P < 0.05, **P < 0.01, ***P < 0.001, ****P < 0.0001.

To investigate why hnRNP A1 knockdown reduces cell proliferation and migration, we performed RNA-seq analysis on sh hnRNP A1 and control (nc) cells. The results showed significant differences in 635 genes, with KEGG analysis indicating a strong association with apoptosis (Fig. 4A). hnRNP A1, an RNA-binding protein, plays a crucial role in cellular regulation. We further explored the role of hnRNP A1 in mRNA regulation through RIP-seq analysis. KEGG pathway analysis revealed that mRNAs bound to hnRNP A1 were also closely associated with apoptosis (Fig. 4B). To determine whether apoptosis is associated with hnRNP A1 knockdown, we examined the expression of apoptosis-related proteins, including Bax and Bcl-2. We found that Bax was significantly upregulated and Bcl-2 significantly downregulated in hnRNP A1 knockdown cells (Fig. 4C–F). We also assessed the difference in apoptotic cell numbers between the two groups using flow cytometry. The results showed that apoptosis levels were significantly higher in hnRNP A1 knockdown cells than in control cells (Fig. 4G, H). Furthermore, studies have shown that hnRNP A1 knockdown reduces BCLXL and XIAP expression in endometrial cancer cells, leading to apoptosis and inhibiting proliferation [8]. To explore the potential signaling pathways regulated by hnRNP A1, we analyzed RNA-seq and RIP-seq data, identifying 344 common genes (Fig. 4I). After applying differential fold-change (FC) filtering to the RIP-seq genes, we conducted a comprehensive analysis with the RNA-seq data. The analysis identified 56 genes with significant overlap, including PPARα mRNA, which showed stronger binding with hnRNP A1 (Fig. 4J, K). Based on RIP-seq data analysis, we predicted potential binding sites for PPARα and hnRNP A1, and identified a region spanning nucleotides 46,235,648 to 46,235,915 within the 3′ untranslated region (3′UTR) of PPARα (Fig. 4L). Dual-luciferase reporter assays showed that hnRNP A1 reduced luciferase activity driven by the PPARα 3′UTR by 35%, indicating that hnRNP A1 can directly bind to the PPARα 3′UTR (Fig. 4M). qRT-PCR and Western blotting analysis revealed that hnRNP A1 knockdown upregulated PPARα expression, indicating that hnRNP A1 regulates PPARα expression (Fig. 4N–P). RIP experiments further confirmed the high binding affinity between PPARα and hnRNP A1 (Fig. 5A). Previous studies have shown that PPARα gene knockout reduces cytotoxic T cell activity, binds to the PD-L1 promoter, and blocks its transcription, inhibiting tumor immune evasion [38]. To verify whether PPARα affects cell proliferation and migration, we conducted a rescue experiment on the hnRNP A1 knockdown group (high PPARα expression group). The results showed that PPARα knockdown rescued the decreased cell proliferation and migration abilities caused by reduced hnRNP A1 expression (Fig. 5B–D). Additionally, apoptotic cell numbers in the nc, sh-hnRNPA1, and si-PPARα 3 groups were assessed using flow cytometry and TUNEL assays. The sh-hnRNPA1 group showed a significantly higher number of apoptotic cells compared to the nc group. However, knockdown of PPARα in the sh-hnRNPA1 group, which exhibited high PPARα expression, reduced apoptosis, although levels remained slightly higher than in the nc group (Fig. 5E–G).

hnRNPs are key regulators of gene expression, playing roles in RNA splicing, transcription, translation control, and maintaining RNA stability [39]. We treated the cells with actinomycin D (Act D) and measured PPARα mRNA levels at different time points to assess its stability. The results showed that PPARα mRNA levels in both cell groups decreased rapidly. Compared to the hnRNP A1 knockdown group, the control group exhibited a faster decline in PPARα mRNA levels and worse stability (Fig. 5H). Besides affecting mRNA stability, ActD can also cause cell cycle arrest, leading to irreversible cell damage. To evaluate whether hnRNP A1 has a protective effect against ActD-induced damage, we measured cell viability at different time points after ActD treatment. The results showed that cell viability in the hnRNP A1 knockdown group rapidly decreased, while the control group maintained better viability (Fig. 5I). Therefore, we conclude that hnRNP A1 influences cell viability by negatively regulating PPARα mRNA stability.

### Lipid accumulation mediated by PPARα is crucial for the growth of CRC cells

**Fig. 6:**
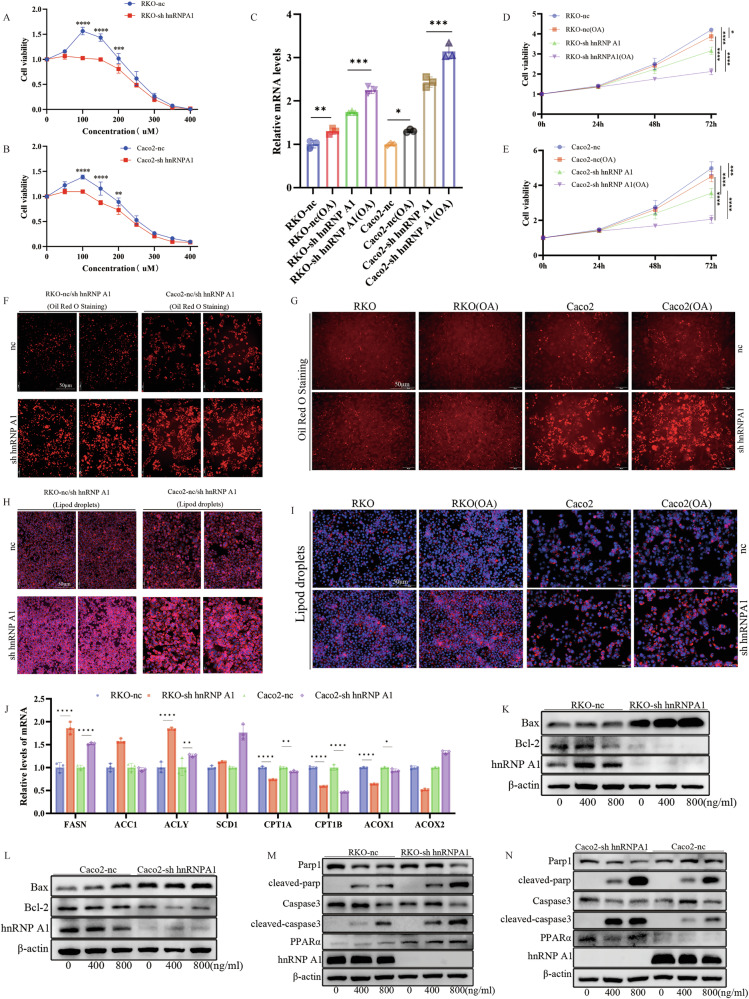
Lipid accumulation mediated by high expression of PPARα is crucial for the growth of CRC cells. **A**, **B** Cell activity at different OA concentrations, *n* = 3 per group. **C** The expression level of PPARα after OA treatment. **D**, **E** Cell viability at various time points after OA treatment, *n* = 6 per group. **F**, **G** Neutral lipid content of RKO (hnRNP A1-nc/sh) and Caco2 (nc/sh) cells and cells after addition of OA (Scale bar: 50 μm), *n* = 3 per group. **H**, **I** Lipid droplet content of RKO (hnRNP A1-nc/sh) and Caco2 (nc/sh) cells and cells after addition of OA (Scale bar: 50 μm). **J** qRT-PCR demonstrates the effect of hnRNP A1 silencing on the expression of FASN, ACC1, ACLY, SCD1, CPT1A, CPT1B, ACOX1, and ACOX2 in CRC cells, *n* = 3 per group. **K**, **L** Western blotting reveals the expression changes of Bax and Bcl-2 expression in hnRNP A1-silenced cells treated with Act D. **M**, **N** Western blotting reveals the expression changes of PARP1, Cleaved-parp, Caspase3, Cleaved-caspase3, PPARα and hnRNP A1 expression in hnRNP A1-silenced cells treated with Act D. *P < 0.05, **P < 0.01, ***P < 0.001, ****P < 0.0001.

Dysregulation of lipid metabolism can affect tumor cell proliferation, survival, migration, and invasion. To investigate whether changes in cell viability are due to PPARα-mediated disruption of lipid metabolism, we conducted studies on lipid metabolism in tumor cells. Free fatty acids, such as oleic acid, are closely associated with fatty acid oxidation (FAO) and lipid metabolism. Studies have shown that oleic acid promotes tumor growth in CRC cells, patient-derived organoids, and mouse CRC models [40], as well as the invasion and metastasis of CRC cells [41, 42]. However, other studies suggest that oleic acid can induce apoptosis and cell cycle arrest, exerting anti-tumor effects [43, 44]. Therefore, we investigated the effect of varying concentrations of oleic acid on cell viability. The results showed that, in the PPARα low-expression group (nc), oleic acid concentrations ranging from 0 to 150 µM promoted cell proliferation, peaking at 100 µM, while concentrations exceeding 200 µM significantly inhibited cell viability. However, the PPARα high-expression group (sh) exhibited reduced tolerance to oleic acid, with a more pronounced effect on cell viability (Fig. 6A, B). Studies have shown that PPARα influences the proliferation and migration of CRC cells, regulates the expression of target genes involved in cell differentiation, immune regulation, and inflammatory responses, and acts as a key regulatory factor in lipid metabolism [38, 45, 46].

Studies have shown that oleic acid regulates lipid metabolism by activating peroxisome proliferator-activated receptors (PPARs), particularly the PPAR-α and PPAR-γ pathways [47–49]. To investigate the effect of oleic acid on PPARα expression, we exposed the cells to different concentrations of oleic acid. The results showed that the PPARα level increased with increasing concentrations of oleic acid (Fig. 6C). Therefore, we treated the cells with 200 µM sodium oleate to assess cell activity. The results showed that after OA treatment, cells with high PPARα expression (sh) exhibited slower proliferation, whereas cells with low PPARα expression (nc) proliferated faster (Fig. 6D, E). We hypothesize that these results may be due to the upregulation of PPARα expression. We then used Oil Red O staining, a lipophilic dye, to observe the differences in neutral lipid content within the cells. The results showed higher lipid content in CRC cells with elevated PPARα expression (Fig. 6F). Therefore, we hypothesize that PPARα may promote specific processes of lipid metabolism, resulting in changes in lipid content. To explore whether OA affects lipid metabolism differently based on PPARα expression levels, we added OA to both cell groups and observed that the PPARα high-expression group (sh) exhibited an enhanced response to OA treatment, while the control group showed no significant change (Fig. 6G). Additionally, we used another lipid droplet dye to confirm this finding and observed a trend similar to that observed with Oil Red O staining. There was a significant difference in lipid content and OA sensitivity between cells with high PPARα expression (Fig. 6H, I). These results confirm the hypothesis that upregulation of PPARα induces lipid accumulation in cells and slows cell proliferation. Therefore, we hypothesize that PPARα is a key factor in lipid metabolism reprogramming in CRC cells, with hnRNP A1 negatively regulating PPARα, thereby affecting lipid metabolic processes and inhibiting cell proliferation and migration.

### Lipid accumulation mediated by PPARα promotes cell apoptosis

Increased fatty acid synthesis, impaired lipid breakdown, and the influence of hormones and signaling pathways contribute to lipid accumulation. Previous studies have shown that PPAR plays a critical role in lipid metabolism and is a key regulator of FAO [50, 51]. To investigate whether hnRNP A1 silencing induces lipid accumulation by stabilizing PPARα mRNA and regulating de novo fatty acid (FA) synthesis, we measured the mRNA expression of key FA synthesizing enzymes, including FASN, ACC1, ACLY, and SCD1, in CRC cells with varying levels of hnRNP A1 expression. We found that FASN and ACLY were significantly upregulated in hnRNP A1 knockdown cells (Fig. 6J). Reduced FAO decreases lipid catabolism, leading to lipid accumulation. To determine whether different levels of hnRNP A1 expression regulate FAO, we measured the expression of key enzymes involved in FAO, including CPT1A, CPT1B, ACOX1, and ACOX2. The results showed that the expression of CPT1A, CPT1B, and ACOX1 was significantly reduced in hnRNP A1 knockdown cells (Fig. 6J), indicating that hnRNP A1 regulates lipid metabolism by controlling the expression of FASN, ACLY, CPT1A, CPT1B, and ACOX1.

**Fig. 7:**
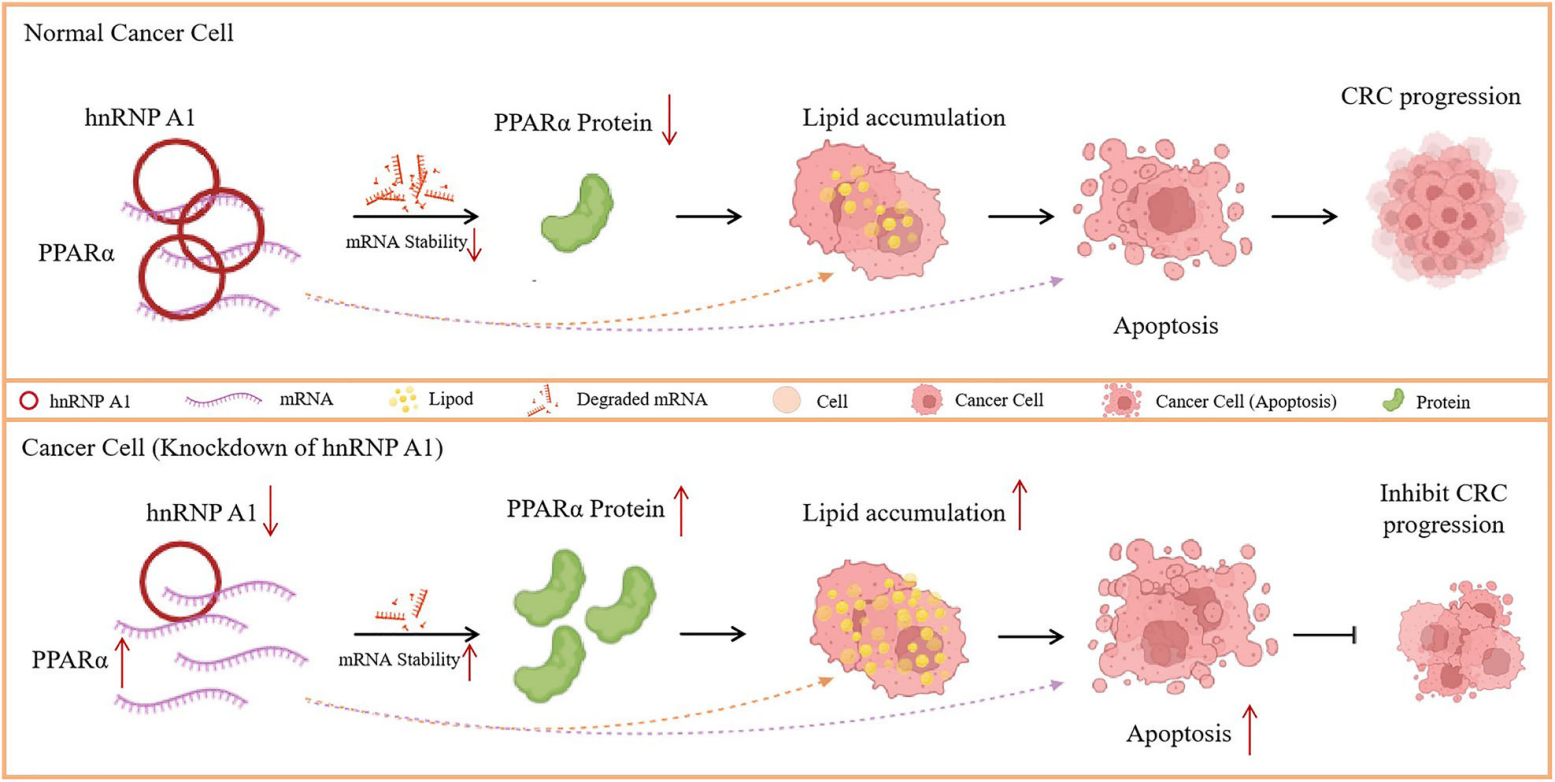
hnRNP A1 promotes cell apoptosis by stabilizing PPARα mRNA and upregulating its expression, which increases neutral lipid content.

Our findings showed that hnRNP A1 negatively regulates the stability of PPARα mRNA. KEGG pathway analysis also revealed a correlation with apoptosis. Therefore, we hypothesize that hnRNP A1 may promote lipid accumulation by regulating PPARα mRNA stability, increasing lipid synthesis, and reducing FAO, thereby promoting cell apoptosis. We first performed Western Blot to measure the expression levels of apoptosis-related proteins in RKO and Caco2 cells. The results showed that after 24 h of Act D treatment at concentrations ranging from 0 to 800 ng/ml, Bax expression increased in a dose-dependent manner. However, Bax expression was higher in SH group cells compared to the NC group, while Bcl-2 expression showed the opposite trend (Fig. 6K, L). Caspase 3 is a key terminal protease in apoptosis. Active caspase 3 (cleaved caspase 3) cleaves various intracellular proteins, leading to cell structure disintegration and cell death. Higher expression levels correlate with a greater tendency for apoptosis [52, 53]. The primary substrate for activated cleaved caspase 3 is PARP, which plays a critical role in DNA damage repair and apoptosis [54]. Cleaved PARP loses its enzymatic activity, destabilizing the cell and accelerating apoptosis. To better observe changes in apoptosis levels, we verified this correlation, and the results showed that the expression levels of cleaved-caspase 3 and cleaved-PARP were positively correlated with apoptosis. We found that their expression levels increased in a dose-dependent manner, similar to Bax expression (Fig. 6M, N). Combining these results confirms that hnRNP A1 promotes lipid accumulation and apoptosis by regulating PPARα mRNA stability. Overall, the regulation of FA metabolism by hnRNP A1 is illustrated in Fig. 7.

## Discussion

CRC is the second leading cause of cancer-related deaths globally. Surgery remains the primary treatment option for long-term survival, but the recurrence rate after surgery is high, and the 5-year survival rate remains low [55]. The prognosis of CRC continues to pose significant challenges. In this study, we analyzed the expression and prognostic significance of hnRNP family members in CRC through database analysis. Our analysis showed that hnRNP family members are highly expressed in CRC and are linked to poor prognosis in patients. Further analysis revealed that hnRNP A1 is significantly upregulated in CRC compared to other family members of hnRNP. High expression of hnRNP A1 is associated with poor OS and PPS in CRC patients, indicating its potential as a prognostic marker. Furthermore, hnRNP A1 is upregulated in various cancer types, consistent with its role as a key oncogene in endometrial cancer [8]. In human CRC tissue samples, the immunohistochemical score of hnRNP A1 was significantly higher in cancer tissue compared to adjacent normal tissue. Additionally, the expression of hnRNP A1 is significantly correlated with TNM staging. hnRNP A1 is upregulated in cancers such as breast and prostate cancer, although its precise function and mechanisms remain unclear. Our findings suggest that targeting hnRNP A1 could offer a promising therapeutic strategy for CRC.

RBPs play a crucial role in biological processes, and their dysfunction can lead to various diseases [56, 57]. The hnRNP family is a large class of RBPs that facilitate the maturation of newly transcribed heterogeneous nuclear RNA into mRNA [39]. Studies have shown that the long non-coding RNA lnc-CTHCC binds to hnRNP K and activates YAP1 transcription, thereby promoting the development of hepatocellular carcinoma [58]. hnRNP K also promotes metastasis by participating in signaling cascades related to the extracellular matrix, cell adhesion, invasion, and angiogenesis [59]. In lung cancer, acetylation of hnRNP A1 regulates the progression of lung adenocarcinoma [60]. In endometrial cancer, Esculetin promotes apoptosis and inhibits tumor cell proliferation by targeting hnRNP A1, which downregulates BCLXL and XIAP [8]. Meanwhile, hnRNPs stabilize and regulate mRNA during its transport. hnRNP A1 is involved in multiple processes, including RNA splicing, transport, degradation, and translation regulation, thereby maintaining mRNA stability [61, 62]. Given its crucial functions, research on hnRNP A1 in various diseases, particularly cancers, has been increasing [63]. Our study shows that hnRNP A1 is essential for the proliferation, migration, and tumorigenic capabilities of CRC cells, both in vitro and in vivo. Although these models have limitations in precisely analyzing CRC onset, they provide valuable insights into the mechanisms of its development.

We also found that hnRNP A1 is involved in apoptosis. Experiments confirmed that dysregulation of hnRNP A1 contributes to CRC development by regulating the expression of apoptosis-related proteins. To further investigate the role of hnRNP A1 in gene expression regulation, we employed RNA-seq and RIP-seq technologies to explore how specific RBPs influence the expression and function of their target mRNAs. The results show that hnRNP A1 significantly interacts with the RNA of the PPARα gene. Interference experiments with PPARα high-expression cells demonstrated that this interaction can rescue cell proliferation, migration and apoptosis. Therefore, we propose that the interaction between hnRNP A1 and PPARα is crucial for the development and progression of CRC. To investigate the underlying mechanisms, we examined the role of hnRNP A1 in maintaining mRNA stability. Actinomycin D (Act D) is a valuable tool for studying mRNA stability over short time periods. It inhibits DNA-dependent RNA polymerase activity, disrupts transcription, and suppresses mRNA synthesis [64–66]. We found that PPARα mRNA stability was significantly higher in the hnRNP A1 knockdown group (sh) compared to the control group (nc), suggesting that hnRNP A1 is crucial for maintaining PPARα mRNA stability. Additionally, Act D exhibits antitumor activity by binding to DNA, inhibiting RNA elongation, which leads to DNA damage, growth suppression, and apoptosis induction [67, 68]. Based on these findings, we hypothesized that mRNA instability in cells is linked to proliferation. Cells were treated with ActD at a specific concentration to assess cell viability, and the results showed that the PPARα high-expression group exhibited lower viability. In conclusion, we propose that hnRNP A1 regulates PPARα mRNA stability, thus influencing cell viability.

To investigate how PPARα influences cell activity, we found that peroxisome proliferator-activated receptors (PPARs) are a class of transcription factors divided into three subtypes: PPARα, PPARδ/β, and PPARγ [69]. PPARα is primarily expressed in tissues involved in fatty acid catabolism and is closely linked to energy homeostasis, immunity, and lipid metabolism [70, 71]. During cancer cell proliferation, alterations in lipid content can impact membrane synthesis, key signaling processes, and cell growth [72]. Studies have shown that fatty acid oxidation (FAO) plays a role in the initiation and growth of CRC cells and is closely linked to drug resistance and metastatic progression [73, 74]. Several enzymes involved in de novo fatty acid synthesis, including FASN, ACLY, SCD, and ACSS2, are abnormally expressed in various cancers, promoting fatty acid synthesis and contributing to malignant tumor phenotypes [75–78]. Additionally, lipid alterations are closely linked to the tumor microenvironment [79–81]. Extensive research has shown that lipid metabolism reprogramming is closely associated with cell apoptosis [82–84]. Our research suggests that knockdown of hnRNP A1 expression leads to increased expression of FASN and ACLY, while decreasing the expression of CPT1A, CPT1B, and ACOX1. Simultaneously, we increased lipid content in the cells by elevating the concentration of OA in the cellular environment. Different concentrations exert varying effects on cell proliferation, with higher concentrations inducing cell death. Additionally, the neutral lipid content in cells from the hnRNPA1 knockdown group was markedly higher than that in the control group. Therefore, we infer that increased fatty acid synthesis and decreased oxidation may cause metabolic imbalance within the cell, promoting cell death. To further explore the relationship between hnRNP A1/PPARα mRNA stability, lipid metabolism, and cell death, we treated cells with various concentrations of ActD and assessed the expression of apoptosis-related proteins. The results indicated that in the hnRNPA1 knockdown group, as the concentration of ActD increased, the expression levels of cleaved caspase 3 and cleaved PARP also elevated. Based on these findings, we infer that hnRNP A1 promotes lipid accumulation and induces cell apoptosis by stabilizing PPARα mRNA.

This study provides compelling evidence for the role of hnRNP A1 in CRC, demonstrating that knockdown of hnRNP A1 expression influences cell proliferation, migration, and other biological processes through lipid metabolism and mRNA stability. Additionally, we have identified PPARα as a novel target for CRC. Despite the intriguing findings, this study has several limitations: (1) The underlying cause of hnRNP A1 upregulation in CRC remains unclear, and further exploration of its mechanisms could offer new opportunities for developing CRC therapeutic strategies; (2) The specific regulatory pathway between hnRNP A1 and PPARα needs further investigation; (3) In vivo experiments tested only the functionality of hnRNP A1 knockdown cells, without additional validation of PPARα.

To further investigate the relationship between hnRNP A1/PPARα mRNA stability, ActD, and lipid metabolism, we treated cells with varying concentrations of ActD and measured the expression of apoptosis-related proteins. The results indicated that ActD increased the expression levels of cleaved caspase 3 and cleaved PARP. Our results showed that as ActD concentration increased from 0 to 800 ng/ml, the expression levels of cleaved caspase 3 and cleaved PARP also increased, with the low hnRNP A1 expression group exhibiting higher levels. These findings suggest that hnRNP A1 inhibits the ActD-induced apoptotic phenotype.

This study provides compelling evidence of the role of hnRNP A1 in CRC, showing that silencing hnRNP A1 affects cell proliferation, migration, and other biological behaviors through fatty acid metabolism and mRNA stability. We also found that ActD exerts its antitumor effects by interacting with the hnRNP A1 protein. Furthermore, it led to the identification of a novel CRC target, PPARα. Despite some interesting findings, this study has several limitations: (1) The cause of hnRNP A1 upregulation in CRC remains unclear, and its underlying mechanism may provide more opportunities for developing CRC therapeutic drugs; (2) The specific regulatory pathways between hnRNP A1 and PPARα need further investigation; (3) In vivo experiments tested only the function of hnRNP A1 knockdown cells, without further validation of PPARα.

## materials and methods

### Public dataset and pathway analysis

TIMER is a comprehensive tool for the systematic analysis of immune infiltration across various cancer types. The TIMER2.0 (timer.cistrome.org) database was used to analyze the expression levels of hnRNP A1 mRNA in pan-cancer and CRC. The relationship between hnRNP expression and the prognosis of CRC patients was analyzed using the Kaplan-Meier Plotter database (kmplot.com). The SangerBox database (sangerbox.com) was used to analyze data related to hnRNP A1 enrichment.

### Materials

VPC-80051 racemate and Sodium oleate were purchased from MCE (USA), fresh VPC-80051 was freshly dissolved in DMSO (D8370, Solarbio, Beijing, China) for each experiment. Primary antibodies against Bcl-2 (12789-1-AP), Bax (50599-2-Ig), β-Actin (66009-1-Ig), PARP1 (13371-1-AP), and Caspase-3/p17/p19 (19677-1-AP) were purchased from Proteintech (Wuhan, China). Primary antibodies against hnRNPA1 (D21H11), Cleaved Caspase-3 (Asp175), and Cleaved PARP (Asp214) (D6X6X) were purchased from Cell Signaling Technology (USA). Primary antibodies against PPARα (EM1707-71) were purchased from HuaBio(Hangzhou, China). Modified Oil Red O Staining Kit(C0158S), Nile Red Staining Kit(C2051S) and One Step TUNEL Apoptosis Assay Kit(C1089) were purchased from Beyotime(Shanghai, China). Actinomycin D was purchased from Aladdin(Shanghai, China).

### CRC cell lines

Human CRC cell lines RKO, Caco2, HT29, HCT116, SW480, and SW620 were purchased from Servicebio (Wuhan, China). RKO, Caco2, HT29, and HCT116 cells were cultured in DMEM (GIBCO, USA), while SW480 and SW620 cells were cultured in RPMI 1640 (GIBCO, USA) medium. The medium was supplemented with 10% fetal bovine serum (FBS; Pricella, China), penicillin (100 U/mL; Servicebio, China), streptomycin (100 μg/mL; Servicebio, China), and Amphotericin B (250 ng/mL; Servicebio, China). All cells were maintained at 37 °C in a 5% CO₂ incubator.

### Human CRC samples

CRC tissue samples were obtained from patients diagnosed at Changhai Hospital in Shanghai. Surgical specimens from 116 CRC patients undergoing radical surgery were collected. These tissues were used to assess mRNA and protein expression levels of relevant genes and to prepare tissue microarrays.

### Apoptosis assay

RKO and Caco2 cells (2 mL) were seeded into a six-well plate at a density of 100,000 cells/mL. After cell adhesion, they were cultured with 1 μg/mL Adarotene (ST1926) (Aladdin, Shanghai, China). The cell pellet was collected after 24 h. The cells were washed twice with PBS, digested using trypsin without EDTA, and collected. After centrifugation at 1500 rpm for 5 min, the supernatant was discarded. The pellet was washed twice with PBS and centrifuged again at the same speed. Finally, the supernatant was discarded, retaining the pellet. Cells were stained using the Annexin V-APC/7-AAD apoptosis kit (Liankebio, Hangzhou, China) according to the manufacturer’s instructions and analyzed for apoptosis using a BD Accuri C6 Flow Cytometer (BD Biosciences, USA). After cell seeding and drug treatment, staining was carried out following the TUNEL assay kit(C1089, Beyotime, Shanghai, China) instructions. After Staining, six randomly selected fields were imaged under a microscope for cell counting, and the average cell count was calculated.

### RNA sequencing (RNA-seq)

RNA-seq was performed following the instructions of the NEBNext Ultra RNA Library Prep Kit for Illumina (New England BioLabs). Briefly, total RNA was extracted from hnRNP A1-depleted or control RKO cells using Trizol reagent. Poly(A) RNA was purified using the PolyTtract mRNA Isolation System and used to generate cDNA libraries. Finally, 2 × 150 bp paired-end sequencing (PE150) was performed on an Illumina Novaseq™ 6000 (LC-Bio Technology Co., Ltd., Hangzhou, China) following the manufacturer’s protocol. The average gene expression values from three independent experiments were used for subsequent analysis.

### RNA immunoprecipitation and high-throughput sequencing (RIP-seq)

Cells were washed twice with ice-cold PBS, collected, and the pellet was resuspended in RIP lysis buffer. Magnetic beads pre-incubated with antibodies were added to the lysate, followed by low-speed rotation and overnight incubation at 4 °C. The magnetic beads were separated using a magnetic separator and washed 4–6 times with the buffer provided in the kit. RNA was eluted and extracted. RT-qPCR was performed according to the instructions of the Magna RIP kit (Millipore, catalog number 17-700) to determine whether the RNA bound to hnRNP A1 was PPARα. For sequencing, rRNA was depleted using the NEBNext rRNA Depletion Kit (New England BioLabs). cDNA libraries were prepared using the NEBNext Ultra RNA Library Prep Kit for Illumina (New England BioLabs) and sequenced on an Illumina Novaseq™ 6000 platform. Each group was sequenced in duplicates.

### Cell viability and migration assays

The Cell Counting Kit-8 (CCK-8)(NCM Biotech, Suzhou, China) was used to assess cell viability. Transwell assays were performed by seeding 8 × 10⁴ cells into the upper chamber without FBS supplementation, while the lower chamber contained 600 μL DMEM supplemented with 10% FBS. After 36–72 h of culture, migrated cells were fixed with 4% paraformaldehyde, stained with Crystal Violet Staining Solution, and counted under a microscope.

### Cell scratch assay

Once the cells formed a confluent monolayer, a “cross”-shaped scratch was created using a 200 μL pipette tip, and the plate was incubated for further culture. At designated time points, the scratch width was observed and imaged under a microscope. The scratch area was measured with ImageJ software to evaluate the cell migration rate.

### Clone formation assay

Cells were seeded into a 6-well plate at a density of 1 × 10³ cells per well. The culture medium was refreshed every 3 days for 14 days. When visible clusters formed, the cells were washed with PBS, fixed with 4% paraformaldehyde, stained with crystal violet, and counted under a microscope.

### Establishment of mouse models

Male BALB/c nude mice (4–6 weeks old) were purchased from Charles River Laboratories, Shanghai. To ensure statistical significance, five samples were randomly selected from each group. Mouse-related studies were conducted in specific pathogen-free facilities, and all procedures were approved by the Laboratory Animal Management Committee of Changhai Hospital, Shanghai, following National Institutes of Health (NIH) guidelines for the care and use of laboratory animals,and all nude mice were randomly divided into groups. To establish a CRC tumor-bearing mouse model, 5 × 10⁶ RKO-nc, RKO-sh hnRNP A1, Caco2-nc, and Caco2-sh hnRNP A1 cells were injected into the armpit. Tumor length was measured with calipers every 2 days. Tumor volume was calculated using the formula: Volume = 1/2 × length × width². At the humane endpoint, the mice were sacrificed, and tumors were excised for imaging and weighing. Tumor xenografts were subsequently stored in liquid nitrogen until IHC analysis.

### Validation of neutral lipids and lipid droplets

To assess the content of neutral lipids and lipid droplets in cells, a Modified Oil Red O Staining Kit(Beyotime, Shanghai, China) and a Lipid Droplets Red Fluorescence Assay Kit(Beyotime, Shanghai, China) with Nile Red were used following the manufacturer’s protocol. Neutral lipids and lipid droplets were stained and imaged using the lipophilic fluorescent dye Oil Red O and the fluorescent probe Nile Red.

### Western blotting

Cells were lysed to extract total protein, which was quantified using the BCA method. Proteins were separated by electrophoresis and transferred onto a PVDF membrane, which was blocked with 5% non-fat milk for 2 h. The membrane was incubated overnight at 4 °C with primary antibody solution, diluted according to the manufacturer’s instructions. The next day, the membrane was washed three times with TBST and incubated with diluted secondary antibody for 1 h at room temperature. After three additional washes with TBST, the membrane was exposed and developed using a chemiluminescent reagent.

### RNA isolation and quantitative real-time polymerase chain reaction (qRT-PCR)

After cultivation, cells were collected, and RNA was extracted using an RNA extraction kit according to the manufacturer’s instructions. The extracted RNA was reverse transcribed into cDNA using a reverse transcription kit. PCR amplification was performed using the synthesized cDNA as a template with a qPCR kit. Relative mRNA expression levels were calculated using the 2^−ΔΔCt^ method.

### Immunohistochemistry (IHC) staining

Specimens were fixed in 4% paraformaldehyde, then embedded in paraffin and dehydrated. Tissue sections (5 μm thick) were baked at 70 °C for 60 min in an oven. Sections were dewaxed in xylene for 10 min and dehydrated using an ethanol gradient, with each step lasting 5 min. Sections were washed in distilled water for 3 min, repeated three times. Sections were incubated with 3% H₂O₂ at room temperature for 10 min, then washed with distilled water and dried. Sections were blocked with 5% BSA solution at 37 °C for 2 h. Primary and secondary antibodies, followed by DAB solution, were applied for color development according to the manufacturer’s instructions. Staining was observed under a microscope and stopped, if appropriate, by washing with tap water. Hematoxylin counterstaining was performed, followed by differentiation in 1% hydrochloric acid alcohol for 3 s and washing with tap water. After dehydration, neutral balsam was used for mounting. Evaluation: Densito Quant software was used for automatic recognition. Dark brown indicated strong positive, brown-yellow moderate positive, light yellow weak positive, and blue nuclei negative. Final scoring was conducted using the H-score method.

### Establishment of stable cell lines and small interfering RNA (siRNA) transfection

hnRNPA1 shRNAs were purchased from GenePharma (Shanghai, China). The virus was added to RKO and Caco2 cells following the manufacturer’s protocol to establish negative controls and stable hnRNPA1 knockdown cells. Cells with successful PTPRO knockout were selected using puromycin (P8230, Solarbio, Beijing, China). The Lipo2000 transfection kit was used to transfect Si-hnRNPA1 and Si-NC (GenePharma, Shanghai, China) into RKO and Caco2 cells following the manufacturer’s instructions. The cells were divided into two groups: the Si-NC group and the Si-PPARα group. Expression analysis was conducted using quantitative real-time polymerase chain reaction (qPCR), and the experiment was repeated three times.

### mRNA stability test

Actinomycin D (ActD) is a polypeptide antibiotic produced by Streptomyces that inhibits DNA-dependent RNA polymerase activity, disrupting the transcription process and inhibiting mRNA synthesis. In this study, cells were treated with a consistent concentration of ActD(MCE, USA), and target mRNA expression was measured at various time points to estimate the half-life of the target RNA, thereby evaluating its mRNA stability.

### Statistical analysis

Statistical analysis of bioinformatics data was conducted using the database’s preset statistical methods. Other numerical data were analyzed with Prism 9.0 and ImageJ software. Measurement data between groups were compared using the *t*-test, while continuous variables were analyzed with analysis of variance (ANOVA). A two-tailed *P* value of <0.05 was considered statistically significant. All experiments were repeated three times.

## Supplementary information

Supplementary File

Supplementary File

## Data Availability

The datasets used and/or analyzed during the current study are available from the corresponding author on reasonable request.
