# Supplementary material for: hnRNP A1 inhibits colorectal cancer tumorigenesis and progression by regulating fatty acid metabolism and RNA stability
Source: Cell Death Discov. 2025 Nov 24;11:542. doi: 10.1038/s41420-025-02814-0 (PMC12644753; doi:10.1038/s41420-025-02814-0)
Supplement: Supplementary file 1 — Supplementary File [file 41420_2025_2814_MOESM1_ESM.docx]

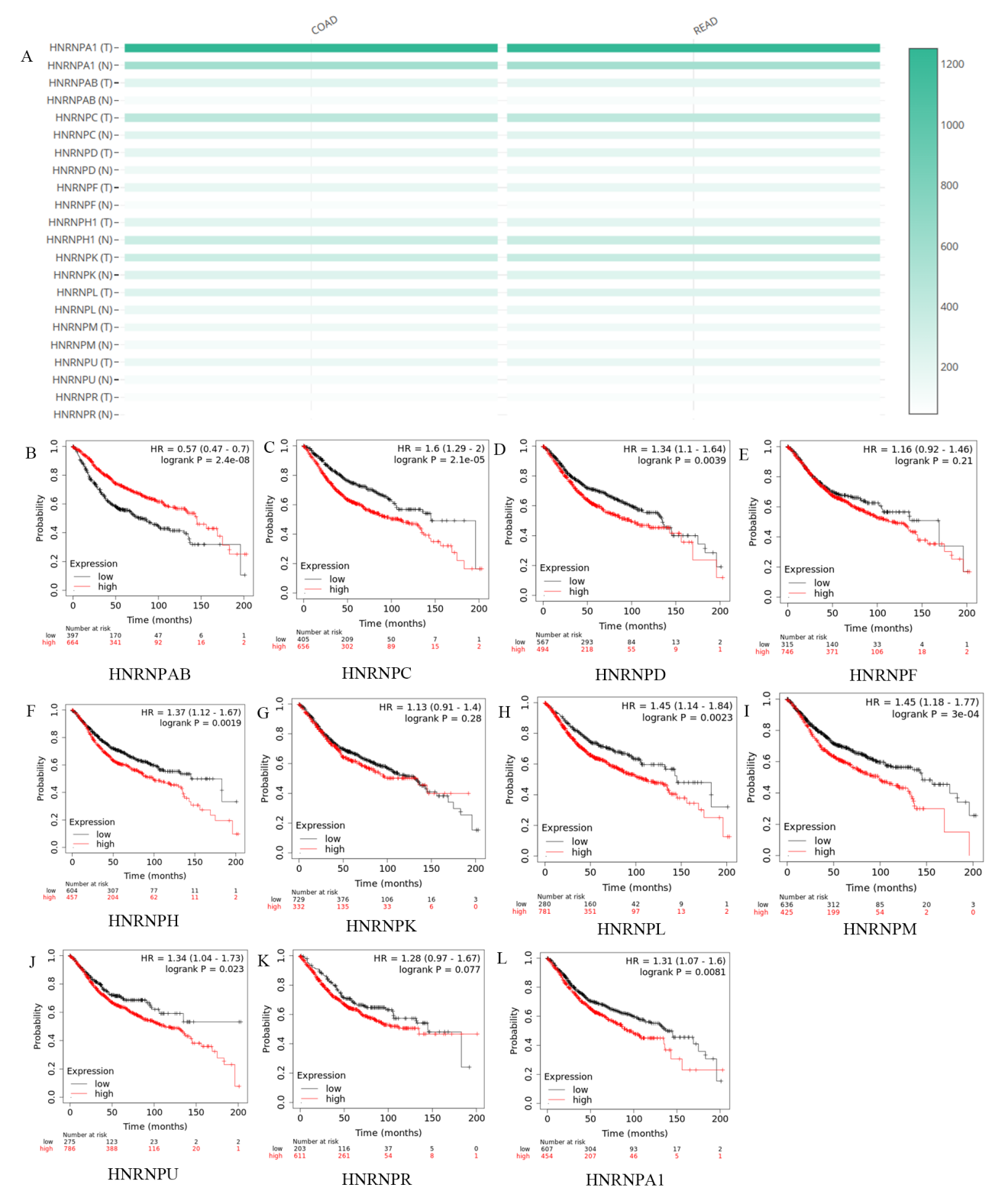


**Supplementary Figure1** Expression of HNRNP family members in colorectal cancer and their association with the patients' prognosis.(A)Expression of HNRNP family members in colorectal cancer.(B-L)Prognostic profile of HNRNP family members in colorectal cancer.


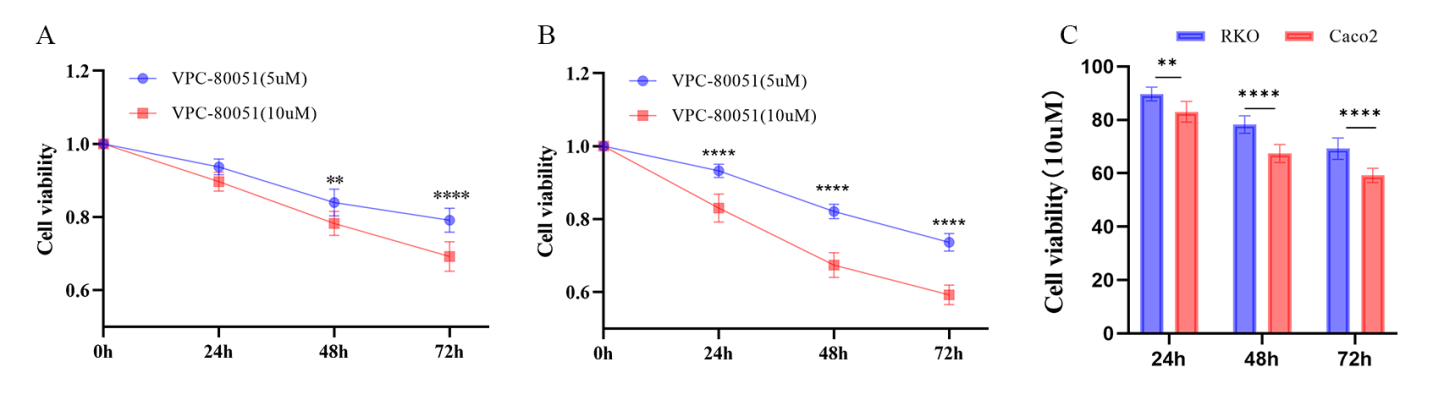


**Supplementary Figure2** Effect of hnrnpa1 inhibitors on cell survival. (A)Effect of hnrnpa1 inhibitors on the survival of RKO cells, n=5 per group.(B)Effect of hnrnpa1 inhibitors on the survival of Caco2 cells, n=3 per group.(C)Viability of the cells treated with 10 μmol/L VPC-80051 at different time points, n=3 per group. **P<0.01, ***P<0.001, ****P<0.0001


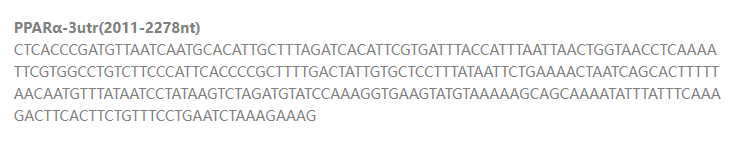


**Supplementary Figure 3** Binding sequences of hnRNP A1 and PPARα
