# Supplementary figures and images for: hnRNP A1 inhibits colorectal cancer tumorigenesis and progression by regulating fatty acid metabolism and RNA stability
Source: Cell Death Discov. 2025 Nov 24;11:542. doi: 10.1038/s41420-025-02814-0 (PMC12644753; doi:10.1038/s41420-025-02814-0)

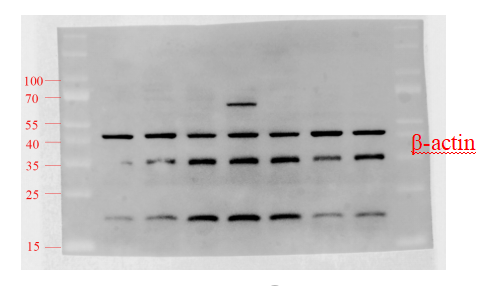
Figure 2A


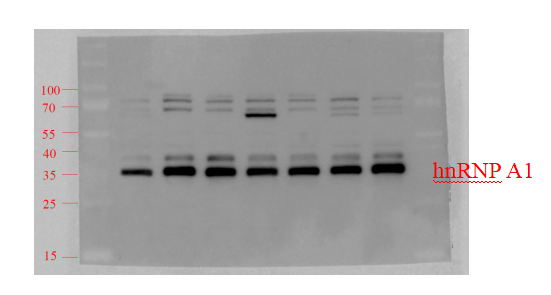


Figure 2D


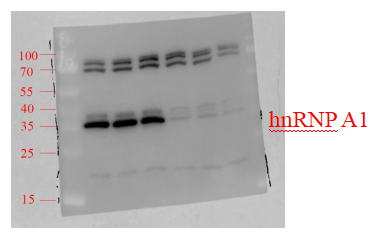

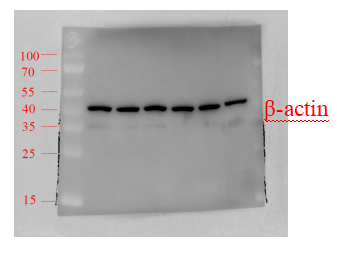


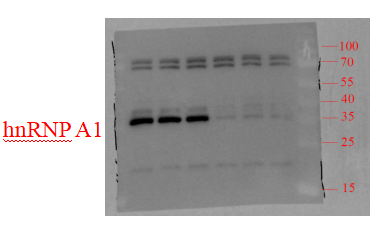

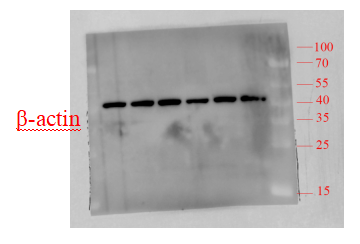


Figure 4C,4E


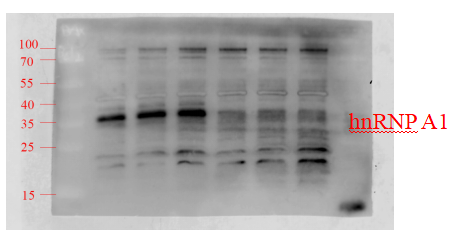

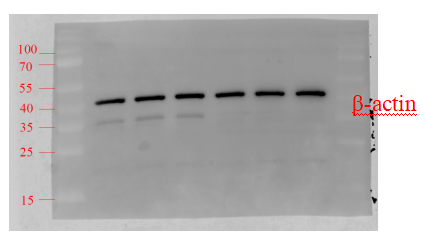


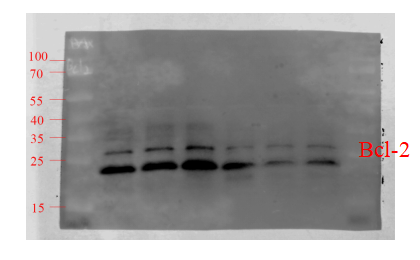

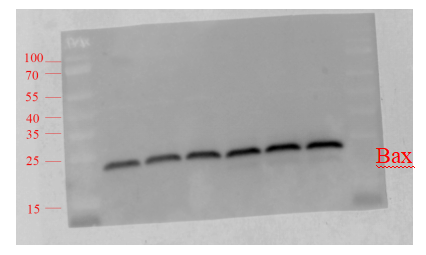


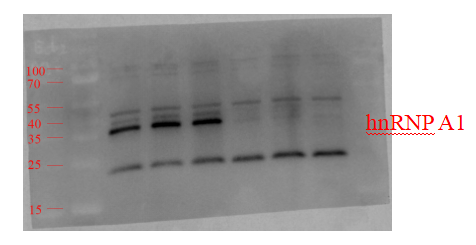

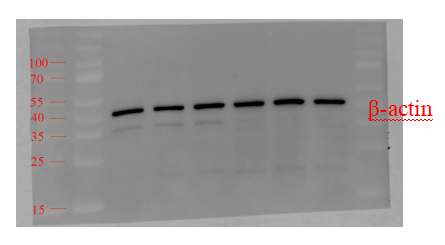


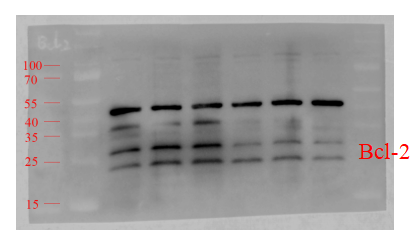

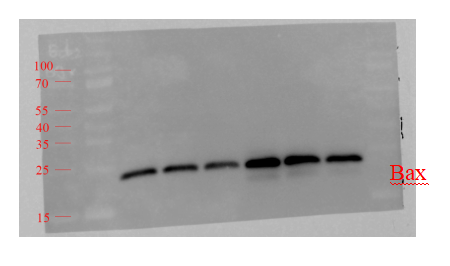


Figure 4O,4P


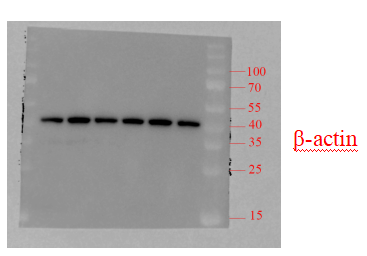

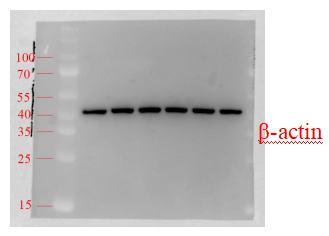


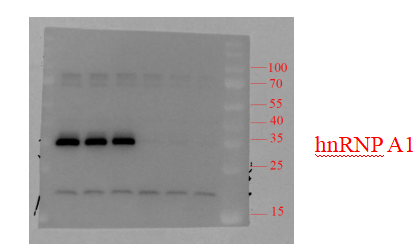

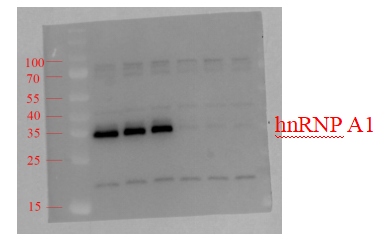


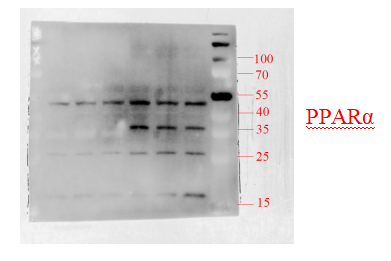

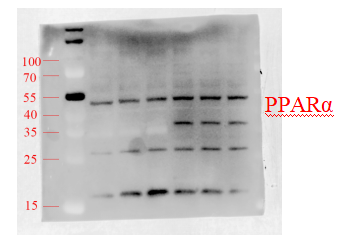


Figure 6K,6L


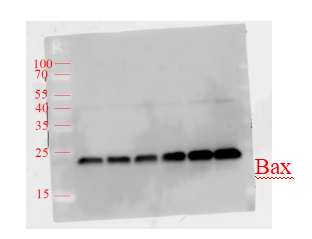

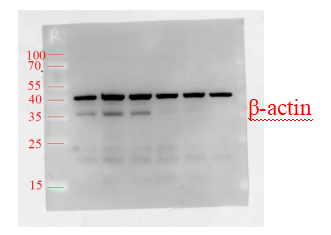


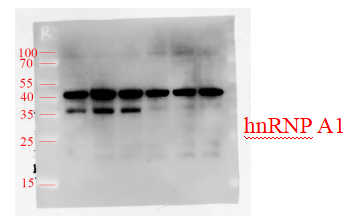


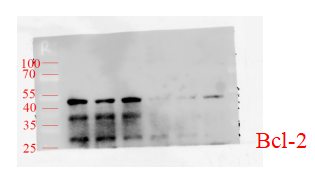


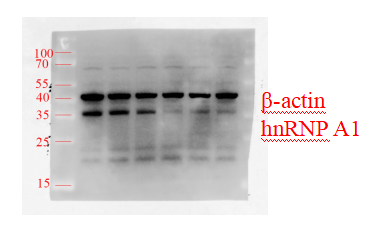

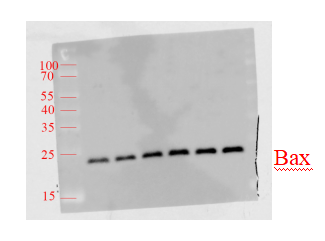


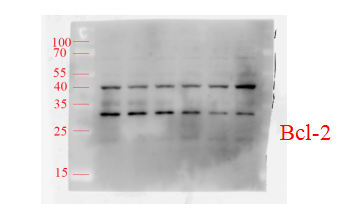


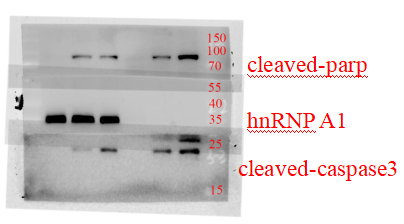
Figure 6M,6N


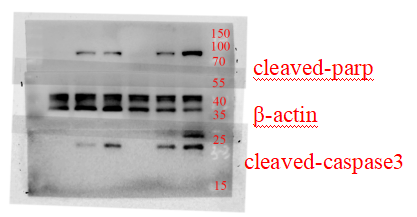


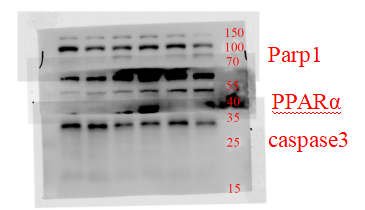

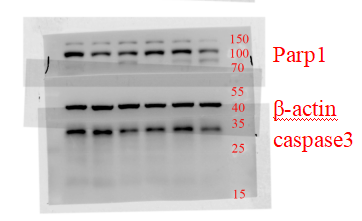


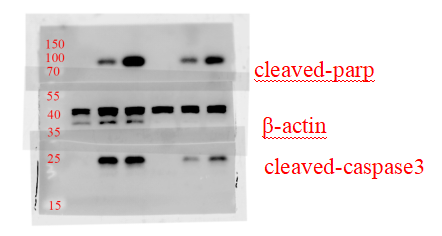


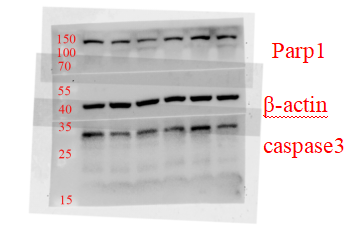


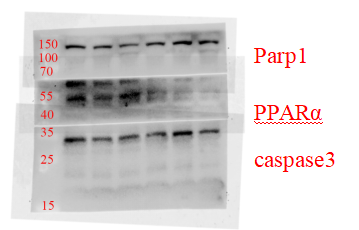


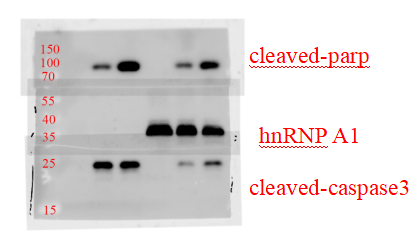

Supplement: Supplementary file 2 — Supplementary File [file 41420_2025_2814_MOESM2_ESM.docx]
